# Interest of Flow Diversion Prostheses in the Management of Unruptured Intracranial Aneurysms

**DOI:** 10.1155/2012/654627

**Published:** 2011-11-03

**Authors:** Xavier Armoiry, Mélanie Paysant, Daniel Hartmann, Gilles Aulagner, Francis Turjman

**Affiliations:** ^1^Pharmacy Department, Groupement Hospitalier Est, Hospices Civils de Lyon, 69500 Bron, France; ^2^Délégation à la Recherche Clinique et à l'Innovation, Cellule Innovation, Hospices Civils de Lyon, avenue Du doyen Lépine, 69500 Bron, France; ^3^UMR-CNRS 5510, Faculté de Pharmacie, Université de Lyon, 69008 Lyon, France; ^4^EA 3452, Faculté de Pharmacie, Université de Nancy, 54001 Nancy, France; ^5^Neuroradiology Departement, Groupement Hospitalier Est, Hospices Civils de Lyon, 69500 Bron, France

## Abstract

Flow diversion prostheses represent a new endovascular approach aimed at treating patients with large wide-neck aneurysms. Our objective is to present this new technology, to review the clinical studies on efficacy, and to emphasize its current limits. Flow diversion prostheses consist of a cylinder made of a large number of braided microfilaments providing a large metallic surface when deployed and inducing a blood flow diversion outside the aneurysm. Two different brands are currently available. Clinical data supporting their efficacy are currently limited to six non comparative cohort studies that included between 18 and 107 patients. Procedural implantation was shown to be feasible in more than 90% and safe with a thirty-day mortality between 2.8 and 5.5%. Complete occlusion rates at twelve months varied between 85.7 and 100%. Even though promising, the current status of flow diversion prostheses needs further evaluation with randomized, prospective, clinical trials with comparison to conventional strategies including endovascular coiling or surgical clipping.

## 1. Introduction

According to a systematic review based on 23 studies, the incidence of unruptured intracranial aneurysm (UIA) is about 2% in the general population, greatly represented by saccular shapes [1]. Whereas individually unpredictable, the risk of rupture is estimated to be 1.2% per year, but it increases up to 6% depending among others on aneurysm diameter or localization [2]. Consequences of rupture are dramatic both in terms of mortality (30–67%) and morbidity (15–30%) [3, 4]. That is why, despite a lack of clinical evidence based on randomized studies [5], it is generally accepted that patients with UIA and major risk factors of rupture should receive a preventive treatment whenever possible [6]. 

Different approaches are then possible [7]. The conventional surgical approach consists of performing a craniotomy followed by a clip ligation, also called microsurgical clipping. Since the nineties, endovascular occlusion using detachable coils, also called coiling, has emerged as an alternative to surgical clipping [8]. Endovascular coiling has been increasingly used because it is thought to be efficient to prevent bleeding with a low rate of complication and minimal invasiveness in comparison to surgical clipping although these alleged advantages versus surgery are not supported by prospective, randomized, clinical trials but only studies with low level of evidence [8]. Furthermore, its long-term efficacy particularly in large wide-neck aneurysm is still debated. Indeed, despite the availability of a wide range of embolization devices and the improvement of procedures using balloon or stent assistance [9, 10], endovascular coiling is hampered mainly because of the high incidence of reopening over time. In a recent meta-analysis, reopening rate was reported at 20%, twelve months after endovascular treatment of large wide-neck UIA, and the rate of retreatment was 10% [11, 12]. Furthermore, the large number of coils implanted may induce a mass effect responsible for the worsening of the patient's condition. In practice, the management of unruptured, large, wide-neck aneurysms remains challenging. 

In this context, a new endovascular technique using flow diverter stents has emerged in the last four years. Our objectives are to present the potential interest and the current limits of this technique in the management of UIA.

## 2. Definition and Presentation of the Technology

Flow diversion prostheses represent a new approach aimed at treating patients with large wide-neck aneurysms. They consist of a cylinder made of a large number of braided microfilaments providing a total metallic surface of up to 30%, when deployed, as opposed to 10% for conventional intracranial stent. Once implanted in the parent artery across the neck, they induce a blood flow diversion outside the aneurismal sac. Since blood flow inside the aneurysm decreases, a progressive thrombosis is observed over time [13, 14]. 

**Figure 1:**
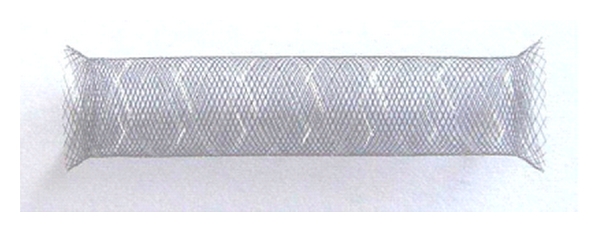
SILK stent (BALT Extrusion Inc.).

Two different brands have been developed over the last four years. The first one is called “SILK” prosthesis (BALT Extrusion Inc.). It is made of 48 braided nitinol and platinium strands that form a high-coverage cylinder once deployed. It exists with different diameters (2 to 5 mm) and lengths (15 to 40 mm) (Figure 1). The SILK stent system includes a self-expanding stent, a delivery system, and a reinforced catheter for its placement. It is repositionable when deployed up to 90%. The SILK stent has received an approval in the European Community. 

**Figure 2:**
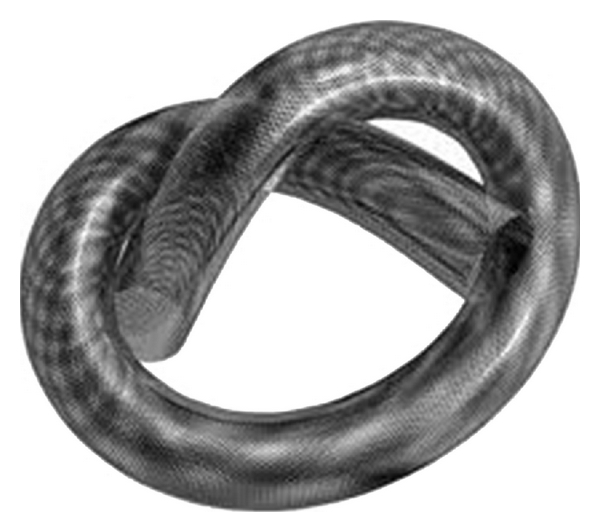
Pipeline embolization device (EV3 Inc.).

The second one is called pipeline embolization device or PED (EV3 Inc.). It is delivered through a standard 0.027-inch internal diameter microcatheter and enables a endovascular “stent-like” construct. The PED has 48 microfilaments made of platinum and nickel-cobalt chromium alloy^. ^(Figure 2). Its diameters are rather similar to the SILK stent (2.5 mm to 5 mm), and the lengths go from 10 to 35 mm. 

The PED has received a European Community and a Food and Drug Administration (FDA) approvals.

The procedures are performed under general anaesthesia and use a transfemoral artery approach. Unexpanded flow diversion stents inserted in the loading device are positioned up the aneurysm and delivered under fluoroscopic guidance. 

Similarly to conventional stenting, patients receive dual antiplatelet therapy (clopidogrel and aspirin) to prevent stent thrombosis prior to the procedure and for at least six months after the procedure. 

This concept of flow diversion stents may be considered as a breakthrough technology since the prostheses enable an intravascular reconstruction in comparison to the intrasaccular approach. 

## 3. Clinical Efficacy and Tolerance Data

### 3.1. Methods

PubMed electronic database was queried in order to search for articles on clinical efficacy and tolerance data. The following key words were used in combination: “*flow diverters stents*,” “*PED*,” “*pipeline embolization device*,” “*SILK stent*,” and “*unruptured aneurysm*.” The FDA website was also consulted. 

### 3.2. Results

Fiorella et al. published the first two experiences reporting a successful use of pipeline embolization device in a total of three patients with either giant sacciform or fusiform symptomatic intracranial aneurysm [13, 14]. Its European Community approval was obtained on the basis of the PITA trial (*pipeline embolization device in the intracranial treatment of aneurysms*). This cohort study was conducted in four centres and included a total of 31 patients with large wide-neck aneurysm (mean size: 11.5 mm, mean neck size: 5.8 mm). Procedural success estimated by successful implantation without immediate complication was 97.9%. The six-month complete occlusion rate was 93.3% [15]. The main complication was the occurrence of two ipsilateral strokes within the 30 days after procedure. 

The FDA approval of PED was recently obtained based on the results of the PUFS (*pipeline for uncoilable or failed aneurysms*) study. This prospective single-arm interventional study included a cohort of 107 patients with large and giant aneurysms (mean size: 18.2 mm, mean neck size: 8.8 mm). Procedural success implantation was 99%. The primary effectiveness endpoint consisting of complete occlusion rate at 180 days without major stenosis was 73.6%. 

The proportion of subjects with major ipsilateral stroke or neurologic death at 180 days after treatment was 5.6%. The PUFS study was then able to show safety and efficacy of PED in large or giant wide-neck aneurysms [16].

The results of two single-centre experiences are also available. A study from Lylyk et al. reported the use of PED in 53 patients treated in the Department of Neuroradiology of Buenos Aires [17]. Forty-four patients were treated by one PED, while 17 and two received two and three PEDs, respectively. In 6% of the cases, endovascular coils were associated. The angiographic complete occlusion rates at six and 12 months were 93% and 95%, respectively. No periprocedural or serious adverse event was observed at 30 days. The rate of minor complications was 11% and mainly consisted in haematoma at femoral puncture site. In the Budapest experience, 18 patients with an important proportion of giant aneurysms were treated with PED. An impressive complete occlusion rate of 94% was observed with a minor incidence of complications. Forty-seven percent of patients received adjunctive coil placement [18]. 

In those cohort studies, more than one stent placement was observed on average, depending on the size of the aneurysm or the level of perioperative blood flow diversion observed on angiography. 

The clinical experiences related to the use of SILK stent are more limited. 

An 18-centre worldwide experience was published on 70 patients presenting an aneurysm and considered as ineligible for conventional endovascular or neurosurgical treatment [19]. The procedures using SILK stent were shown to be feasible with an implantation success of 96% and an acceptable mortality rate of 2.8% with regards to the high proportion of large or giant aneurysm. At 12 months, an impressive complete occlusion rate of 100% was observed, but long-term followup yet concerns a limited number of patients. 

Another experience of Lubicz and colleagues is published on 29 patients considered not eligible to other endovascular approaches (47% of fusiform or circumferential shape aneurysm) [20]. The procedural implantation success was 90% with a mortality rate of 2.8%. Angiographic followup available at three or six months reports a complete occlusion rate of 69%. 

The six cohort study experiences are summarized in Table 1. Comparisons between flow diversion prostheses are not possible not only because criteria for patient selection were not similar, but also because endovascular procedures were different in terms of adjunct coil placement.

## 4. Discussion

### 4.1. Current Limits

The theoretical interest of flow diversion stents is to enable endovascular reconstruction when conventional approaches, mainly represented by endovascular coiling or surgical clipping, are not technically feasible or not appropriate because of a major risk of morbidity or recurrence over time. It is expected to relieve neurological symptoms due to mass effect and to decrease aneurismal recurrences over time. Even though promising and representing a breakthrough technology, the current knowledge on flow diversion prostheses is still limited, and their respective positioning in routine practice must be further evaluated. 

First, questions may be raised on the technical procedure by itself. More precisely, there is currently no guideline regarding the number of stents needed to be implanted and the interest of adjunctive coil placement. The number of stents to implant will first depend on the aneurismal neck. In very large or giant aneurysm, the length of one flow diverter stent may be not sufficient to cover all the aneurysm, and therefore, several stents may be required. For example, Fiorella and colleagues reported the use of seven serial telescoped PEDs to bridge a 29 mm diameter aneurysm [14]. The level of immediate blood flow diversion expected after stent placement is not clear, and therefore, the need to further add a second stent inside the first one (“in-stent” stenting) to increase blood flow diversion can be discussed. Table 1 shows that the number of stents required per aneurysm greatly varies between studies, and it seems to be dependent on each neuroradiologist's experience. 

The second debate is on whether any adjunctive coil placement should be necessary. 

Recently, Turoswki and colleagues reported an early fatal haemorrhage due the aneurysm rupture after vascular reconstruction using the SILK stent without any coils associated [21]. Obviously, it is impossible to know if adjunctive coil placement could have prevented this rupture. If so, would one, two, or more coils be necessary? Until more clinical data is available, this question is still debated. 

The current positioning of flow diversion prostheses in the strategy is also to be evaluated. 

Firstly, clinical experiences reporting the use of flow diverters are limited to noncomparative cohort studies including between 18 and 107 patients. Thus, there is currently no head-to-head comparison of flow diversion prostheses versus other conventional approach, including endovascular coiling or surgical clipping in large wide-neck aneurysm in terms of long-term angiographic and clinical outcome. 

Furthermore, flow diversion prostheses are expected to improve quality of life of patients due to the suppression of mass effect-associated symptoms, but no comparative data is currently available versus endovascular coiling. 

A long-term view on tolerance is needed to accurately determine the real incidence of secondary rupture or the risk of stent thrombosis. Whereas certainly rare, this latter complication is possible as illustrated by Fiorella et al. concerning a very late thrombosis in a patient treated with PED [22].

At last, taking into consideration their substantial cost (approximately €10,000 per stent in Europe, i.e., about US $14,000) and the number of stents required per patient, the economic impact of flow diversion stents should be further evaluated through cost-benefit analysis.

### 4.2. Perspectives

Several randomized clinical trials are now ongoing to improve knowledge on this technology.

Taking into consideration that most of patients with large wide-neck aneurysm are suitable for conventional approach, the randomized FIAT trial (*flow diversion in intracranial aneurysm treatment) *will compare the clinical outcome between flow diversion and best standard treatment including endovascular coiling or surgical clipping [23]

In patients for whom an endovascular treatment is decided, three clinical trials are ongoing or planned. A first one called COCOA (*complete occlusion of coilable intracranial aneurysms) *is being conducted in the United States to compare PED versus endovascular coiling in small wide-neck aneurysm [24]. In Europe, the MARCO POLO trial is comparing the SILK prosthesis reconstruction versus endovascular coiling in large wide-neck aneurysm [25]. The EVIDENCE trial is about to compare the PED versus endovascular coiling (with or without balloon or stent assistance) in large wide-neck aneurysm in a prospective, multicentric, randomized study. This French trial promoted by Lyon University Hospital will be aimed at evaluating the two strategies both in terms of clinical and economical endpoints in order to measure the incremental cost-effectiveness ratio of PED versus endovascular coiling.

## 5. Conclusions

Endovascular reconstruction using flow diversion prostheses is a highly innovative technique for the treatment of patients with UIA. Whereas promising, many questions still remain regarding this technique by itself and its current positioning in the strategy. Flow diversion prostheses are indicated in rare clinical situations such as giant or fusiform aneurysm in which no acceptable therapeutic approach exists, and their interest in the management of large aneurysm should be evaluated prospectively with comparison to conventional techniques, including endovascular coiling or surgical clipping.

## Figures and Tables

**Table 1:** Summary of clinical trials conducted on flow diversion stents.

Device	SILK stent (BALT)		Pipeline embolization device (EV3)			
Number of centres	3	18	4	10	1	1
Number of patients	29	70	31	107	53	18
Number of aneurysms	34	70	31	107	63	19
Number of small/large/giant aneurysms	18/12/4	18/37/15	20/9/2	0/85/22	33/22/8	5/10/4
Procedural success implantation	90%	96%	97. 9%	99%	97%	
Adjunctive coil placement	0%	14%	51.6%	1%	6%	47%
Number of stent implanted per patient	1.42	/	1.35	3.1	1.37	2.16
30 days mortality rate	4%	2.8%	0	2.8%	0	5.5%
3- or 6-month complete occlusion rate	69%	50%	93%	81.8%	93%	94%
12-month complete occlusion rate	/	100%	NA	85.7%	95%	/
Reference	[20]	[19]	[15]	[16]	[17]	[18]
